# Is NAD(P)H quinone oxidoreductase 1 a tumor promoter or suppressor in gastric cancer?

**DOI:** 10.3389/fonc.2023.1143108

**Published:** 2023-04-28

**Authors:** Lei Cao, Yuanqin Chen, Shuangta Xu, Hongwei Cheng

**Affiliations:** ^1^ Department of Pathology, Xiang’an Hospital of Xiamen University, Xiamen, China; ^2^ Department of Pathology, Quanzhou Women’s and Children’s Hospital, Quanzhou, China; ^3^ Department of Thyroid and Breast Surgery, The Second Affiliated Hospital of Fujian Medical University, Quanzhou, China; ^4^ School of Public Health, Center of Molecular Imaging and Translational Medicine, Xiamen University, Xiamen, China

**Keywords:** NAD(P)H quinone oxidoreductase 1, gastric cancer, prognosis, biomarker, immune checkpoint blockade

## 1 Introduction

A great deal of evidence has been reported on NAD(P)H quinone oxidoreductase 1 (NQO1) as an important regulator in some tumors. However, the role of NQO1 is still unclear, NQO1 might function as either a pro-oncogenic or a tumor suppressor gene. This study focuses on the expression and the clinical significance of NQO1 in gastric cancer, the comprehensive evaluation of NQO1 of which we believe is conducive to an in-depth exploration of clinical value and provides a reference for subsequent basic research and translational applications.

A retrospective study reported that NQO1 was overexpressed in gastric cancer and associated with poor prognosis, which demonstrates that NQO1 might be a promising biomarker in the prognostic evaluation for gastric cancer patients with adjuvant chemotherapy (1). However, some concerns should be discussed, and we also provided some supplementary results about NQO1 in gastric cancer. We believe that the comprehensive understanding of NQO1 expression and its association with patient outcomes and the potential exploration of NQO1 as a prognostic and therapeutic target have a translational value.

## 2 NQO1 is a specific gene expressed in gastric tissues

Previous publications showed that NQO1 has diametrically opposite functions in different tumors. NQO1 was reported to be overexpressed in breast cancer (2), lung cancer (3), colon cancer (4), and pancreatic cancer (5, 6), which were closely associated with tumor occurrence and development. The biological analysis also demonstrated that NQO1, as a tumor promoter, could enhance tumor proliferation and metastasis and induce drug resistance to chemotherapy (7). On the other hand, NQO1 also performs as a scavenger of superoxide. NQO1 may play as a tumor suppressor in this sense (8). Thapa et al. reported that NQO1 showed a decreased expression in prostate tumor tissues, and the knockdown of NQO1 could promote the tumorigenesis of prostate cancer (9). Notably, the overexpression of NQO1 in gastric cancer was also reported (10), and the alteration of NQO1 gene C609T polymorphism was associated with increased gastric cancer risk (11). However, the clinical significance of NQO1 was little known, and the full study of NQO1 in gastric cancer is an insightful work.

**Figure 1:**
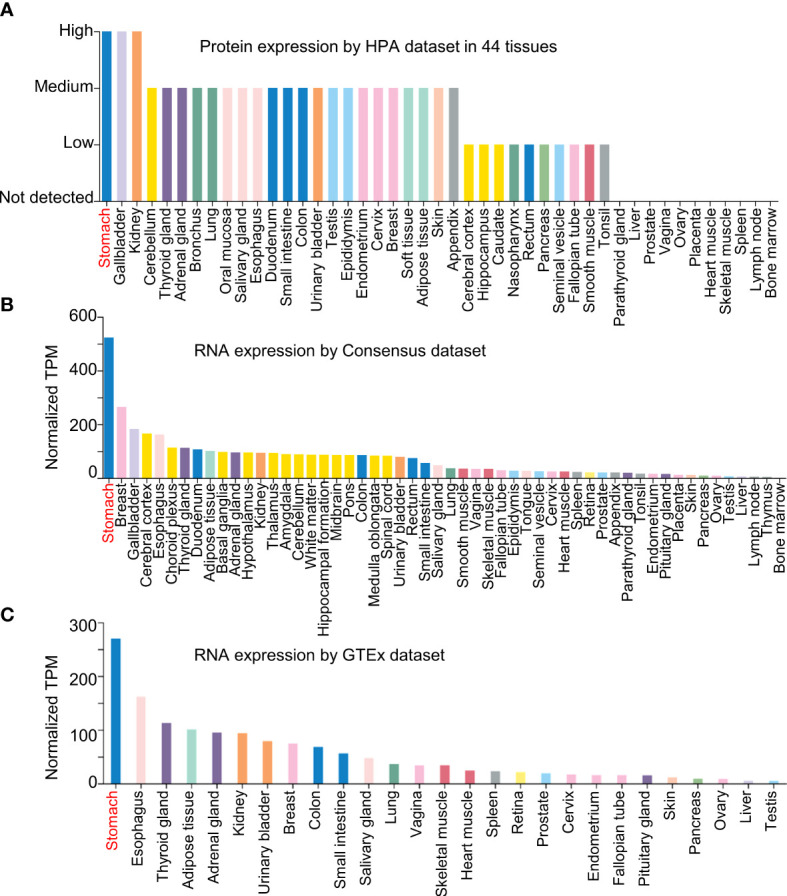
NQO1 is a specific gene expressed in gastric tissues. **(A)** The NQO1 protein expression level was assessed using Human Protein Atlas dataset in 44 tissues. **(B, C)** The NQO1 transcript level was conducted in Consensus dataset and GTEx dataset.

In Jiang’s report, NQO1 was expressed in AGS cells, a gastric cancer cell line (1). We believe that the comparison between normal gastric epithelial cells (such as GES-1) and tumor cell lines with different histological grade, well-differentiated N87 gastric carcinoma cell line, or poorly differentiated BGC-823 cells could be included in the evaluation of NQO1 expression in gastric cancer cells, and we believe that the comparison could fully demonstrate the expression status of NQO1 in gastric cancer. To fully understand the expression of NQO1 in gastric cancer, we analyzed the expression of NQO1 in different tissues by the Human Protein Atlas, Consensus, and Genotype Tissue Expression (GTEx) datasets in the Human Protein Atlas (12). As revealed by the results, NQO1 protein was highly expressed in stomach tissues (**Figure 1A**), and similar results were also confirmed in an RNA expression analysis by Consensus and GTEx datasets (**Figures 1B, C**). The consistent results demonstrated that the expression of NQO1 in stomach tissues was the highest than in other tissues, suggesting that NQO1 was specifically expressed in gastric tissues. Therefore, the remarkable expression in gastric tumor tissues could not support the conclusion of NQO1 overexpression in gastric cancer. In Jiang’s report, the overexpression of NQO1 in gastric cancer tissues was also not confirmed. The authors showed the NQO1 protein expression by immunohistochemical staining, and they explored the association between NQO1 and some pathological features. Unfortunately, the statistical analysis also showed no significant difference between gastric cancer patients with high NQO1 and those with low NQO1 expression. Then, we proposed a question on the expression status of NQO1 in gastric cancer.

## 3 Decreased NQO1 is a favorable prognostic biomarker for gastric cancer

**Figure 2:**
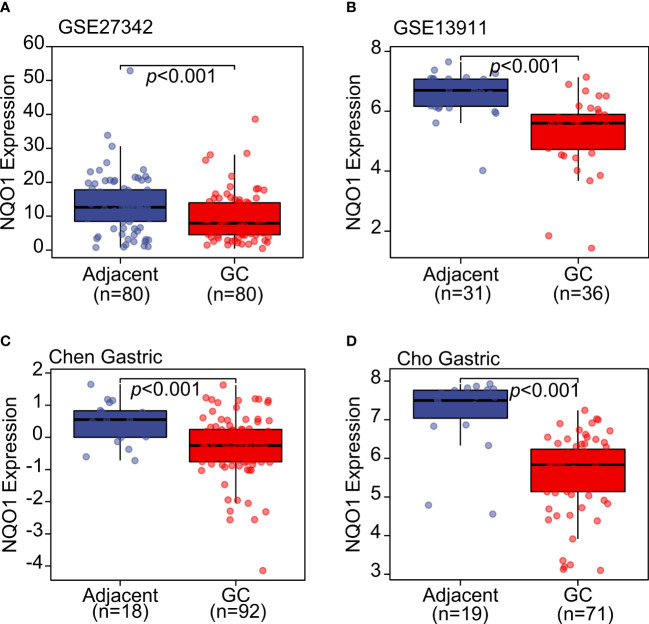
The NQO1 transcript level was downregulated in gastric cancer tissues compared with adjacent normal tissues. **(A)** GSE27342 and **(B)** GSE13911 datasets were included to evaluate the NQO1 transcript level. **(C)** Chen Gastric and **(D)** Cho Gastric datasets from Oncomine database were applied to analyze the expression level of NQO1 in gastric tumor tissues and adjacent tissues.

In order to overcome the remarkable individual difference of patients with gastric cancer, several datasets were included in the analysis as shown in **Figure 2**. GSE27342 (13) and GSE13911 (14) both showed a significantly decreased gene expression of NQO1 in gastric cancer tissues than in adjacent normal tissues (**Figures 2A, B**). Furthermore, the Oncomine database, including Chen Gastric and Cho Gastric datasets, was also used to test the NQO1 expression difference between gastric tumor tissues and gastric normal tissues (15). The consistent results also confirmed that NQO1 gene expression was downregulated in gastric cancer tissues (**Figures 2C, D**). In this sense, our analysis based on the gene level of NQO1 in gastric cancer was diametrically opposite to Jiang’s report. Considering other previous reports that NQO1 was overexpressed in gastric tumor tissues than in normal gastric mucosa tissues and the specific expression of NQO1 in stomach tissues (**Figure 1**), the comprehensive validation of the expression status of NQO1 in gastric cancer is an important work.

Given the discovery of reduced NQO1 expression in gastric cancer, it is necessary to re-examine the correlation between NQO1 expression and patients’ survival. In Jiang’s work, the gastric cancer patients with high NQO1 expression showed poor overall survival probability, suggesting that NQO1 was a tumor promoter. Considering the opposite results about the expression of NQO1 in gastric tumor tissues, we further analyzed the clinical significance of NQO1 in gastric cancer. The results from the Kaplan–Meier survival analysis tool (16) showed that three transcript isoforms of NQO1 (including 201467_s_at, 201468_s_at, and 210519_s_at) all demonstrated that the gastric cancer patients with a higher expression of NQO1 had a favorable overall survival (OS) probability (**Supplementary Figures S1A-C**). Furthermore, similar results were also validated in a post-progression survival (PPS) analysis (**Supplementary Figures S1D–F**). The total number of included gastric cancer patients was significantly larger than those of some previous reports (1, 10), and we believe that it could more objectively reflect the clinical significance of NQO1 in gastric cancer. Another point worth noting is that Jiang’s report on gastric cancer patients with surgery only showed that the higher-NQO1 patient groups displayed a higher OS rate, which was similar to our findings (**Supplementary Figure S1**). Therefore, Jiang’s results from all patients were inconsistent with the results from patients with surgery only.

To explore the impact of NQO1 on the survival of patients who underwent surgery only, gastric cancer patients who received surgery-only treatment were included in the OS and PPS analysis. The findings indicated similar results to those obtained from studies including patients who received various treatments (**Supplementary Figure S2**). Furthermore, we also confirmed the conclusion in SurvExpress portal (17). As shown in **Supplementary Figure S3**, the gastric cancer patients of The Cancer Genome Atlas program were divided into high-risk or low-risk groups according to the survival status, and the patients with a high risk showed a significantly lower NQO1 expression in gastric tumor tissues.

## 4 Discussion

Based on the evidence presented, it can be concluded that our results are consistent with and support the notion that the decreased level of NQO1 in tumor tissues predicts the poor prognosis for gastric cancer patients. Moreover, the evidence suggests that NQO1 may function as a tumor suppressor in the context of gastric cancer. Then, regarding the inconsistency in Jiang’s results, we speculate that the number of samples might be the most significant factor. More importantly, we also need to consider what other factors may have contributed to the opposite findings between Jiang’s report and our study. We propose two hypotheses. Firstly, potential post-transcriptional regulation might contribute to the inconsistent results between the gene and protein levels. Secondly, the heterogeneity of gastric cancer might lead to the significant difference in the results of these studies.

Another important issue is that Jiang’s work showed that gastric cancer patients with a high NQO1 expression were more sensitive to 5-fluorouracil-based adjuvant chemotherapy, indicating that NQO1 may play a role in chemotherapy sensitization. It is a very interesting phenomenon that deserves further investigation.

In conclusion, assessing the prognostic value of NQO1 in patients with gastric cancer who have undergone adjuvant chemotherapy is a crucial aspect of clinical practice. However, a further analysis of NQO1 expression in gastric cancer and its correlation with patient prognosis is necessary to fully understand the clinical implications of NQO1. It is also essential for researchers to conduct more comprehensive studies on the molecular mechanism of NQO1 in gastric cancer and explore its potential as a prognostic and therapeutic target for translation into clinical practice.

## Author contributions

LC and YC conducted the results collection. LC and HC wrote the manuscript. HC and SX revised the manuscript. All authors contributed to the article and approved the submitted version.
