# Supplementary material for: Is NAD(P)H quinone oxidoreductase 1 a tumor promoter or suppressor in gastric cancer?
Source: Front Oncol. 2023 Apr 28;13:1143108. doi: 10.3389/fonc.2023.1143108 (PMC10178065; doi:10.3389/fonc.2023.1143108)
Supplement: Supplementary file 1 [file DataSheet_1.docx]

Supplementary Material

NAD(P)H quinone oxidoreductase 1 is a tumor promoter or suppressor in gastric cancer?

Lei Cao^1†^, Yuanqin Chen^2†^, Shuangta Xu^3^*, Hongwei Cheng^4^*

^1^ Department of Pathology, Xiang'an Hospital of Xiamen University, Xiamen 361002, China

^2^ Department of Pathology, Quanzhou Women’s and Children’s Hospital, Quanzhou 362000, China

^3^ Department of Thyroid and Breast Surgery, The Second Affiliated Hospital of Fujian Medical University, Quanzhou 362000, China

^4^ School of Public Health, Center of molecular imaging and translational medicine, Xiamen University, Xiamen 361002, China

*** Correspondence:**Hongwei Cheng
[hongwei1026@hotmail.com](mailto:hongwei1026@hotmail.com)

Shuangta Xu
[xushuangta@fjmu.edu.cn](mailto:xushuangta@fjmu.edu.cn)

† These authors have contributed equally to this work and share first authorship

# Supplementary Figures


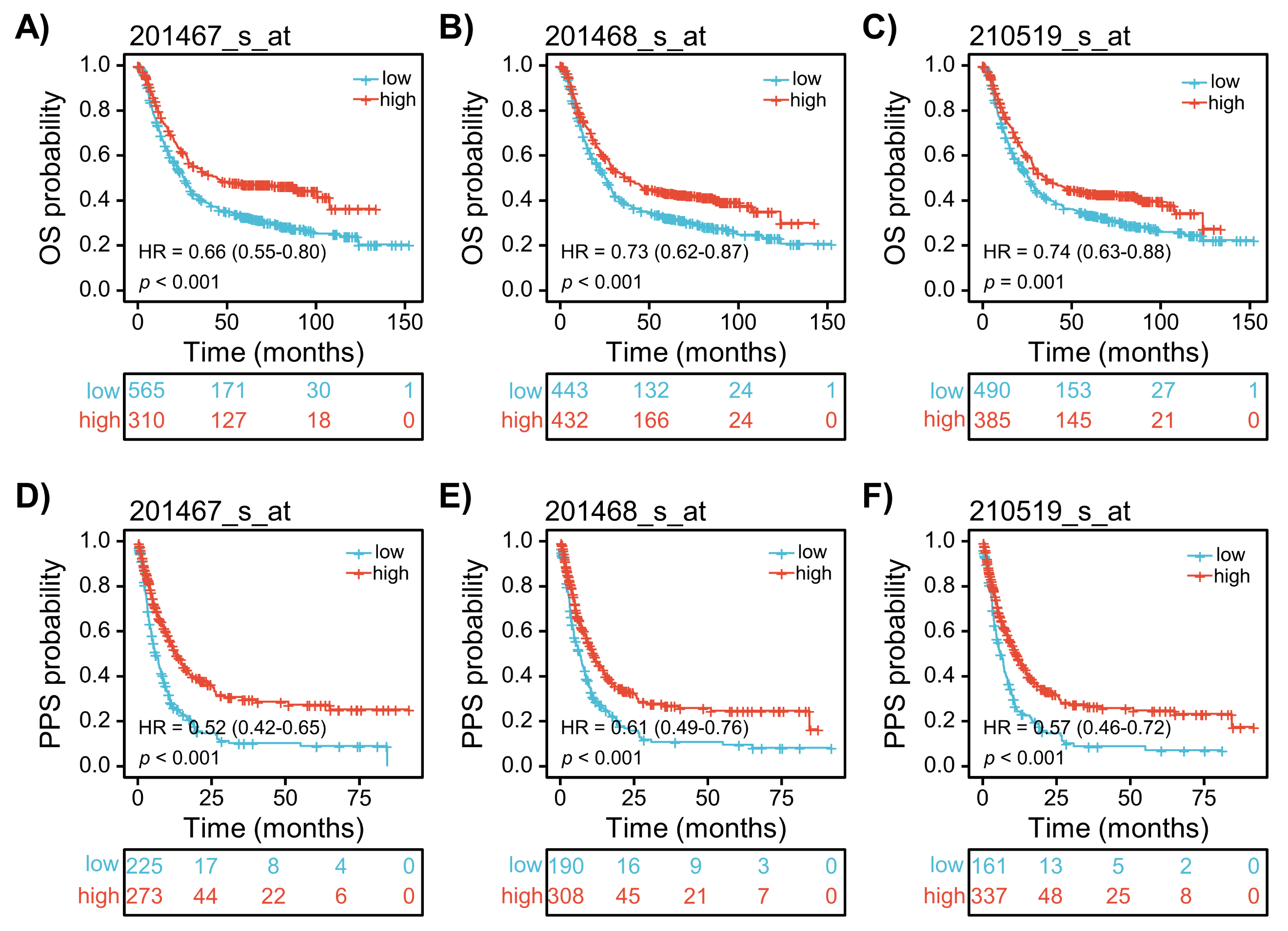


**Supplementary Figure 1.** The lower expression of NQO1 predicted the poor prognosis for gastric cancer. A: The gastric cancer patients were divided into high or low NQO1 groups by the median level of NQO1 transcript isoform 201467_s_at, B: 201468_s_at and C: 210519_s_at, and then subjected to the OS survival analysis; D: The gastric cancer patients were divided into high or low NQO1 groups by the median level of NQO1 transcript isoform 201467_s_at, E: 201468_s_at and F: 210519_s_at, and then subjected to the PPS survival analysis.


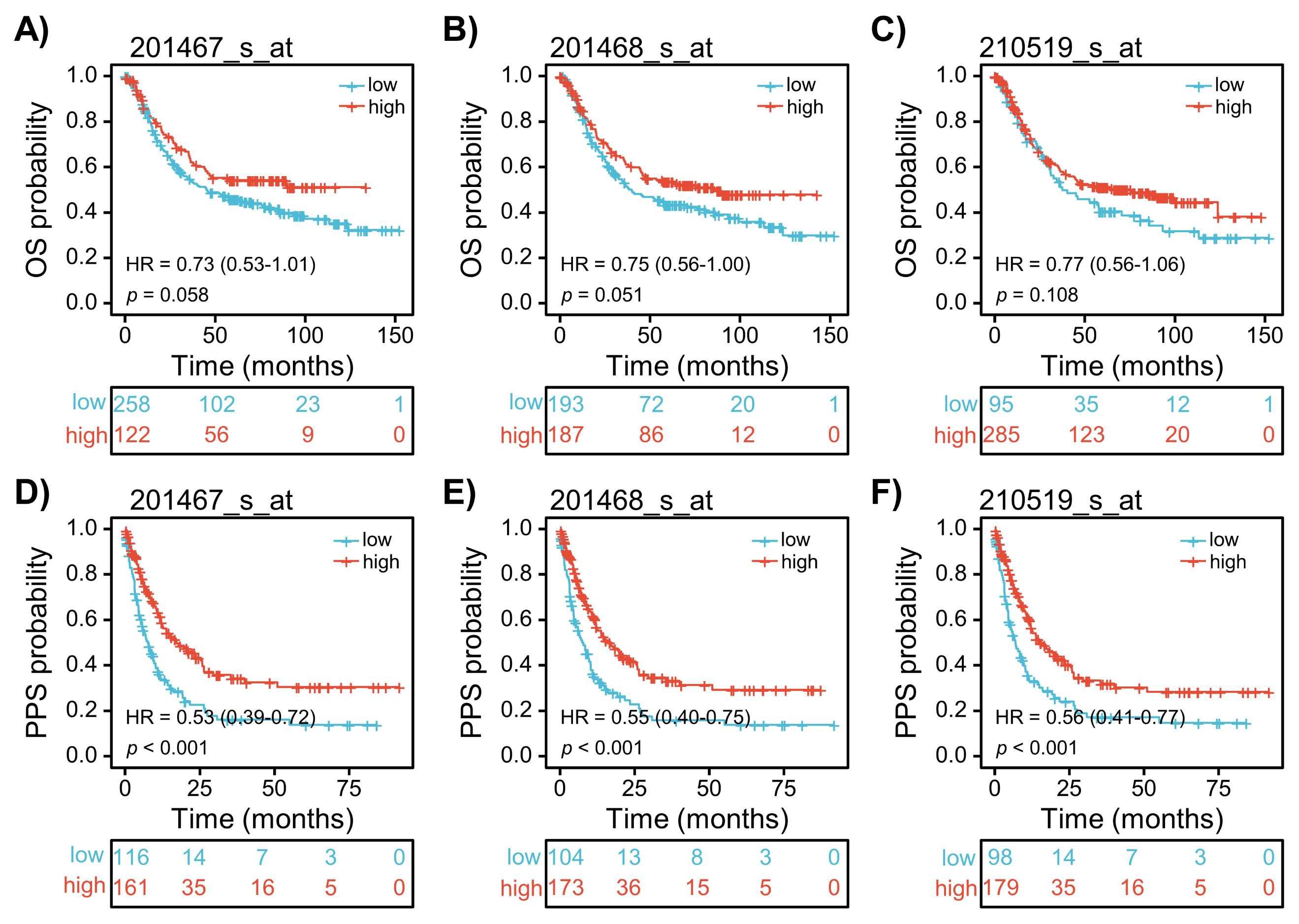


**Supplementary Figure 2.** Lower NQO1 as an unfavorable prognostic biomarker for gastric cancer patients with surgery treatment only. A: The gastric cancer patients with surgery treatment only were divided into high or low NQO1 groups by the median level of NQO1 transcript isoform 201467_s_at, B: 201468_s_at and C: 210519_s_at, and then subjected to the OS survival analysis; D: The gastric cancer patients with surgery treatment only were divided into high or low NQO1 groups by the median level of NQO1 transcript isoform 201467_s_at, E: 201468_s_at and F: 210519_s_at, and then subjected to the PPS survival analysis.


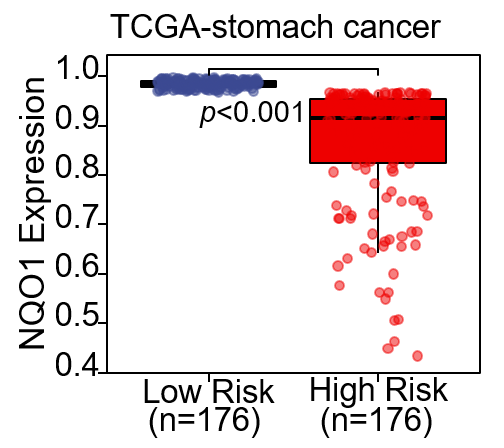


**Supplementary Figure 3.** The gastric cancer patients with high risk showed the decreased NQO1 expression. The survival data from TCGA dataset in SurvExpress portal was subjected to the comparison of NQO1 expression level of gastric cancer patients with high risk or low risk.
